# Multiscale Modeling of a Chain Comprising Selective Laser Melting and Post-Machining toward Nanoscale Surface Finish

**DOI:** 10.3390/ma16247535

**Published:** 2023-12-06

**Authors:** Reza Teimouri

**Affiliations:** Chair of Production Engineering, Faculty of Mechanical Engineering, Cracow University of Technology, John Pawła II, 31-864 Cracow, Poland; reza.teimouri@pk.edu.pl

**Keywords:** selective laser melting, milling, surface rolling, surface roughness

## Abstract

The generation of rough surfaces is an inherent drawback of selective laser melted (SLM) material that makes post-treatment operation a mandatory process to enhance its surface condition and service performance. However, planning an appropriate and optimized chain to attain the best surface finish needs an integrated simulation framework that includes physics of both additive manufacturing and post-processing. In the present work, an attempt is made to model the alternation of surface roughness which is produced by SLM and post-processed by milling and sequential surface burnishing. The framework includes a series of closed-form analytical solutions of all three processes embedded in a sequence where the output of the preceding operation is input of the sequential one. The results indicated that there is close agreement between the measured and predicted values of arithmetic surface roughness for both SLM material and the post-processed ones. It was also found that a nanoscale surface finish is obtained by finishing milling and single pass rolling at a static force of 1500 N. In addition, the results of the simulation showed that elimination of the milling process in the chain resulted in a six-times-longer production time that requires three times bigger rolling force compared to a chain with an included milling operation.

## 1. Introduction

A rough surface and poor structural integrity are known as the main drawbacks of materials which are produced by SLM. Post-processing including thermal, mechanical, and thermo-mechanical treatments and their combination are known as plausible methods to enhance the surface condition of additively manufactured materials through removing surface anomalies, refining the microstructure, and generating compressive residual stress [1]. The selection of the appropriate post-processing techniques and their sequences needs knowledge about the properties of additively manufactured materials to be produced and characteristics of post-processing treatments.

Machining, as a mechanical surface treatment, is usually used for enhancing the surface roughness of an SLM material through removing the rough surface layer. Nonetheless, it has great impact on the quality of the surface’s finish and does not significantly change the mechanical and metallurgical aspects of the surface integrity. Therefore, other mechanical non-metal removal post-processing methods like peening and rolling (and their different alternatives) are used as a sequential treatment after machining [2].

Surface rolling and its similar alternatives like burnishing have been extensively utilized for the post-processing of SLM material. The process can be either used as a sequential treatment immediately after the SLM process or as a downstream process after machining. There are several research efforts which have used a chain additive manufacturing process followed by machining for surface property enhancement of metals produced by additive manufacturing.

Rotella et al. [3] used a post-processing chain heat treatment, turning, and burnishing to analyze the fatigue life and surface integrity of samples produced by laser powder bed fusion. They found that rolling speed and force have a great impact on surface integrity and fatigue life. Teimouri et al. [4] applied a surface rolling process to enhance the roughness of stainless SLM parts. They reported that the surface roughness can be significantly reduced by increasing rolling depth up to a certain level. Zhang and Liu [5] used sequence of turning and burnishing for property enhancement of laser clad material Cr-Ni-based stainless steel. They showed that the surface integrity of the material processed in the chain depends on the initial condition of the material before reaching the chain’s last operation burnishing. Varga et al. [6] applied sliding friction burnishing for property enhancement of Ti-6Al-4V fabricated by selective laser melting. They revealed that the final surface roughness of post-processed samples depends on the surface roughness of the as-built material. Sayyadi et al. [7] revealed that burnishing can be used as a final post-treatment operation after selective laser melting and shot peening to enhance the fatigue life of stainless steel 316. They confirmed that the sequence of shot peening–burnishing yields better roughness compared to solely shot peening or burnishing. Zhang et al. [8] applied warm ultrasonic surface rolling followed by heat treatment for additively manufactured Fe-based layers. They proved a significant improvement in states of residual stress, porosity, and hardness compared to as-cladded material and the one processed in cold conditions or warm conditions without heat treatment.

Raaj et al. [9] applied burnishing for the post-processing of alloy 718 built by electron beam additive manufacturing. They revealed that superior surface integrity (roughness, hardness, and residual stress) is obtained when burnishing is used after grinding of the as-built material. Yaman et al. [10] applied surface rolling for surface integrity enhancement of Inconel 718 produced by SLM. They showed that increasing the rolling force from 250 N to 750 N significantly enhances the microhardness and wear resistance of an SLM material. However, the improvement of surface hardness was not that significant as the single-pass burnishing process was carried out on an SLM material. Hao et al. [11] used ultrasonic surface rolling for post-treatment of AM Inconel 718 fabricated by high-speed laser cladding. They proved that surface hardness and wear resistance are greatly improved following ultrasonic-surface rolling process. Sunny et al. [12] used interlayer surface rolling for enhancement of the residual stress state of Inconel 625 fabricated by SLM. They reported residual stress changes from tensile to compressive type for SLM material following the strategy of SLM + burnishing.

All the above-reviewed research used experimental approaches to assess the surface integrity evolution of materials produced by SLM and post-processed by mechanical treatments. However, the chain design based on this experimental approach is costly, time-consuming, and full of uncertainties. To overcome this problem, the chain should be simulated through a series of mathematical expressions where the output of a process is used as an input of sequential one. Numerical Simulation based on the finite element method (FEM) has been widely used for the modeling of additive manufacturing processes including SLM or direct energy deposition [13,14]; however, the method is really time demanding and makes process optimization challenging. Moreover, applying FEM for the simulation of a chain is too complicated as the output of the downstream process needs to be used as the input of the upstream one. However, simulation based on closed-form analytical models provides means to effectively identify the physics of the variations in a chain, as well as to optimize it in terms of process quality characteristics and production time. Therefore, in the present work, an analytical framework is developed to simulate the surface roughness alternation of an SLM material 3D printed by the SLM process and post-treated by milling and surface rolling. In this study, we are going to identify how the chain can be optimized by adjusting the parameters of each operation to attain the minimum surface roughness, subjected to a high production rate.

## 2. Materials and Methods

This section includes two main parts; in the first part, the simulation framework which was used for the modeling of the surface roughness is described in detail. In the second part, the experimental approach for confirmation of the developed simulation model will be explained.

### 2.1. Simulation Framework

In order to simulate the surface roughness evolution in the chain starting with selective laser melting and followed by milling and burnishing, mechanics of the surface generation of each process should be firstly identified through series of analytical formulations. Then, based on their sequence in a chain, the surface roughness value of the upstream operation will be considered as the initial value of the downstream one.

#### 2.1.1. Analytical Modeling of Surface Generation in Selective Laser Melting

**Figure 1:**
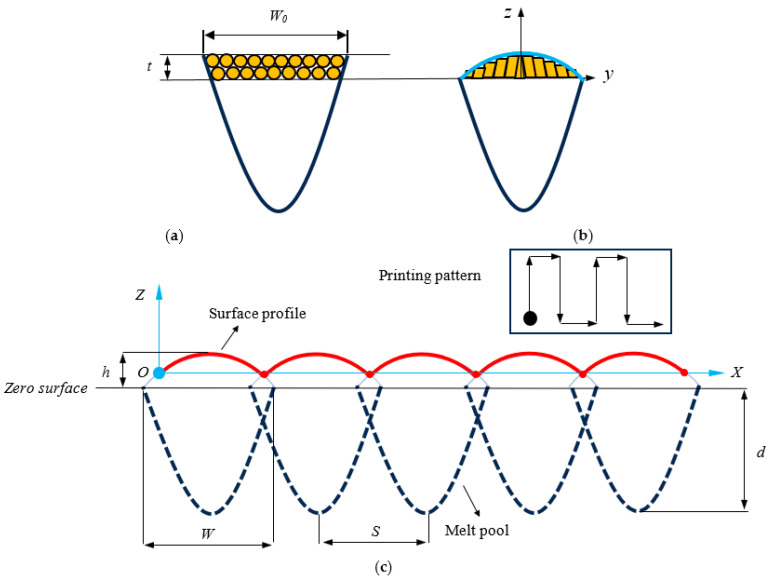
Schematic diagram of (**a**) melt pool in melting stage, (**b**) formed cap over the melt pool surface after solidification, (**c**) roughness generation through duplication of caps formed on melt pool surface following 3D-printing pattern.

As a strong and proven assumption, surface roughness generation of the selective laser melting process is mainly attributed to the formation of the caps on the top of each solidified melt pool as result of surface tension, as shown in Figure 1a,b. Then, it is duplicated over the surface through 3D printing which moves following a zig-zag pattern, as shown in Figure 1c. According to the figure, the surface profile is a function of melt pool width (*W*), melt pool height (*h*), melt pool depth (*d*), and hatch spacing (*S*). The melt pool geometry itself is a function of the distribution of temperature of the laser beam over the volume of bounded powder. It was proved in different studies [15,16,17] that a point moving heat source derived from general convection diffusion problems and simplified and solved taking into account the steady-state condition can describe well the distribution of temperature from a laser heating source.

(1) $$T(x,z)=\frac{P\eta }{4\pi k\sqrt{x^{2}+z^{2}}}exp\left[\frac{-V\rho C\sqrt{x^{2}+z^{2}}}{k}\right]+T_{0}$$

where *T* is the temperature of each point in *xz* plane, *T*_0_ denotes room temperature, *P* is the heating source power, *η* is the powder absorption coefficient, *V* is the printing in *y* direction, *k* denotes the heat conduction coefficient, and *ρ* is the density of the material.

In order to calculate the melt pool geometries, the melting temperature should be embedded in Equation (1). Accordingly, the met pool depth (*d*) is calculated by solving the following non-linear equation at *x* = 0 [4].

(2) $$\left[2\pi k\left(\frac{T_{m}-T_{0}}{P\eta }\right)\right]d+exp\left[\left(\frac{V\rho C}{2k}\right)d\right]=0$$

By calculating the melt pool depth, the profile of the melt pool underneath the zero surface that is in shape of a paraboloid can be calculated using following equation.

(3) $$z=x^{2}-d$$

The equations describe the characteristics of the melt pool in the melting stage. However, in order to calculate the roughness values, the melt pool geometries should be modified after solidification while a hump is formed on the top surface of the solidified melt pool. According to previous studies, it was reported that the depth and width of the melt pool remains unchanged after solidification. Accordingly, in order to calculate the size of the hump, mass conservation law is applied to the volume of the melt pool after and before solidification. It was also proved that considering the hump profile as part of the ellipse will bring accurate results compared to experimental values, while printing stainless steel 316L [16,17]. Therefore, the height of the melt pool is calculated using the following formula.

(4) $$\left\{\begin{array}{c} h=\frac{4}{W\pi }\left[Wt+\int_{\frac{W}{2}}^{\frac{W_{0}}{2}}\left(x^{2}-d\right)dx\right]\\ W_{0}=2\sqrt{d}\\ W=2\sqrt{d-t} \end{array}\right.$$

where *t* stands for layer thickness and *W*_0_ denotes powder band width.

Once the equation of the cap and height of the melt pool are identified, the surface roughness generation profile (as shown in Figure 1c) is derived using the following expression.

(5) $$Z=\left(\frac{2H}{W}\right)\left\{\sqrt{W^{2}-{\left[X-\left(i-1\right)s\right]}^{2}}-\sqrt{W^{2}-\frac{s^{2}}{4}}\right\}\;\;\;i=1,2,\ldots ,n$$

#### 2.1.2. Analytical Modeling of Roughness Alternation by Milling

In this work, a sequence of post-processing methods including milling and burnishing is designed for enhancing the surface roughness of the 3D-printed material.

Here, firstly the surface generation of the milling process is modeled using the principle of the face milling process. By having the *Rz* value of the surface that is generated after the SLM process (which equals to cap height *Rz*), the axial milling depth of the cut in face milling conditions can be identified. Accordingly, based on the values of H, the milling depth of cut, and milling cutter inserts, the new milling surface is generated. Considering that the face milling cutter insert has a squared profile with a nose radius of *r_ε_* and approach angle of α, the engagement of the milling cutter and the surface results in generation two new surface conditions.

**Figure 2:**
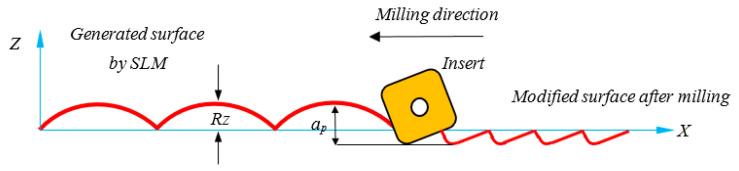
Schematic diagram showing modification of roughness of SLM surface after milling.

**Figure 3:**
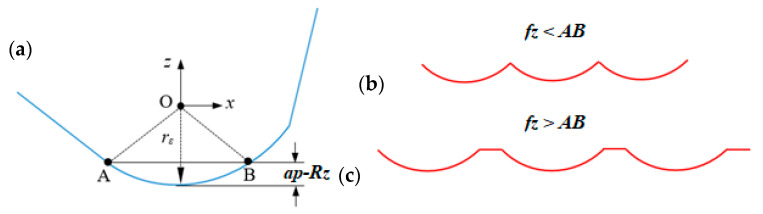
(**a**) A sketch of engagement of cutting insert and workpiece in finished milling process, (**b**) surface generation profile when the feed rate is less than tool–workpiece engagement width, (**c**) surface generation profile when the feed rate is greater than tool–workpiece engagement width.

During the milling of an SLM material’s surface, in most cases, the surface roughness can be finished by only a single-pass machining process since the maximum height of the roughness is usually less than the maximum allowable depth of cut. Accordingly, the modified roughness profile after milling (as shown in Figure 2) depends on the height of roughness from the previous operation (i.e., Rz_0_ of SLM sample), milling axial depth of cut (*a_p_*), geometry of cutting insert (cutter angles), and the value of feed per each cutting insert (*fz*). When the difference between the depth of cut and SLM roughness (i.e., *a_p_* − *Rz*) is less than the tool nose radius (*r_ε_*), the roughness profile depends on the values of feed per cutting insert. Then, a series of intersected circles with straight lines between them are developed, as shown in Figure 3. Here, the tool workpiece engagement length *AB* is defined as follows:

(6) $$\bar{AB}=2\sqrt{\left(a_{p}-Rz\right)\left(2r_{\varepsilon }-a_{p}+Rz\right)}$$

Accordingly, when *f_z_* is smaller than *AB*, the surface profile includes a series of circles (as shown in Figure 3b) which can be calculated using the following equation.

(7) $$Z_{i}\left(x\right)=r_{\varepsilon }-\sqrt{r_{\varepsilon }^{2}-{\left(X-if_{z}\right)}^{2}},\;\;\;\;\frac{\left(2i-3\right)f_{z}}{2}\leq X\leq \frac{\left(2i-1\right)f_{z}}{2}\;$$

When *f_z_* is greater than *AB*, the surface profile includes a series of circles and straight lines between them (as shown in Figure 3c).

(8) $$Z_{i}\left(x\right)=\left\{\begin{array}{c} r_{\varepsilon }-\sqrt{r_{\varepsilon }^{2}-{\left(X-if_{Z}\right)}^{2}},\;\;\left(i-1\right)f_{Z}-\sqrt{\left(a_{p}-Rz\right)\left(2r_{\varepsilon }-a_{p}+Rz\right)}\leq X\leq (i-1)f_{Z}+\sqrt{\left(a_{p}-Rz\right)\left(2r_{\varepsilon }-a_{p}+Rz\right)}\\ r_{\varepsilon }-a_{p}+Rz,\;\;\;\;\;\;\;\;(i-1)f_{Z}+\sqrt{\left(a_{p}-Rz\right)\left(2r_{\varepsilon }-a_{p}+Rz\right)}\leq X\leq if_{Z}-\sqrt{\left(a_{p}-Rz\right)\left(2r_{\varepsilon }-a_{p}+Rz\right)}\;\; \end{array}\;\;\;\;\right.$$

where *I* is the counter that can be set *i* = 1, 2, …, *n* based on the number of surface profiles.

#### 2.1.3. Analytical Modeling of Roughness Alternation by Burnishing

As a final finishing process, based on the characteristic of the roughness and properties of the material, burnishing can be used either immediately after production by SLM or after milling. In this work, through developing a simulation model of the chain, we are going to identify either if it is possible to use burnishing immediately after selective laser melting to minimize the time of production, and if so how many pass numbers are required to achieve the desirable roughness; or if a milling process is required to achieve the desired roughness.

**Figure 4:**
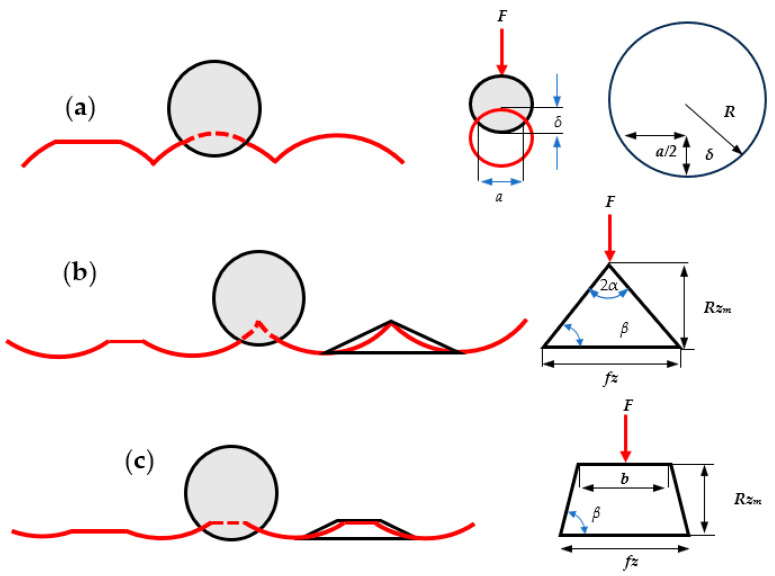
Contact of roller with roughness profiles generated from preceding operation, (**a**) SLM process, (**b**) milling process condition #1, (**c**) milling process condition #2.

As burnishing is used on the rough surface, the principle of contact of a rigid solid roller with a rough surface is used to model the alteration of the roughness profile after burnishing. The deformation of every individual roughness which is generated by each operation (SLM-induced roughness and milling-induced roughness under condition #1 and #2) is different when they are subjected to the first rolling pass. According to the geometry of contact shown in Figure 4a, the deformation of the roller with roughness induced by SLM follows the contact of two cylinders. Here, in order to obtain the deformation depth and width, i.e., *δ* and *a*, the principle of contact mechanics of two cylinders should be applied. Accordingly, during the plastic deformation of two cylinders in the rolling contact process, the force corresponding to plastic deformation can be obtained using the following equation [18].

(9) $$F_{R}=\bar{P}aL$$

where *F_R_* denotes the amount of load which is applied on every individual roughness that corresponds to the number of roughness which is being deformed by each roller (*N*) and can be calculated by dividing the contact width of the cylinder and flat surface based on the Hertz equation and scan speed during SLM, as expressed in Equation (10). *P* is the average contact pressure which equals 3*σ_s_* [18], *a* is the contact width, and *L* is the contact length equal to the length of the roller. Accordingly, the relationship between the contact depth and contact length (as shown in Figure 4a) can be obtained based on geometry considering neglection of the second order of small terms [18] using Equation (11).

(10) $$F_{R}=\frac{F_{n}}{N},\;\;\;\;\;N=round\left[\frac{\sqrt{\frac{4RF_{n}}{\pi LE_{eq}}}}{s}\right],\;\;\;\;F_{n}=\frac{F}{n}\;,\;\;\frac{1}{E_{eq}}=\frac{1-\vartheta_{1}^{2}}{E_{1}}+\frac{1-\vartheta_{2}^{2}}{E_{2}}$$

(11) $$\delta =\frac{a^{2}}{8R}\;\;,\;\;\;\;a=\;\frac{F_{R}}{3\sigma_{s}aL}$$

By identifying the depth of the contact, the modified surface roughness height after the first rolling pass can be obtained by the difference of the SLM roughness height and plastic deformation depth, i.e., *Rz_b_* = *Rz*_0_ − *δ*.

In burnishing a milled surface profile, depending on the type of roughness (as shown in Figure 3), the deformation during rolling is different. While surface rolling a milled surface with surface profile #1, it is assumed that the intersected circles make a wedge as shown in Figure 4b [19]. Then, the surface profile is altered by deformation of this wedge and calculating the corresponding plastic deformation depth. According to the previous research, the plastic deformation depth and corresponding roughness can be calculated using the following equation.

(12) $$\left\{\begin{array}{c} \delta =\frac{2F_{n}f_{z}}{\sqrt{2r{Rz}_{m}}L\left(2\alpha +\sin2\alpha \right)E_{eq}}\ln\frac{\pi E_{eq}L}{\left(2\alpha +\sin2\alpha \right)\sigma_{s}}-\frac{\sigma_{s}}{E_{eq}}\left(\frac{2F_{n}f_{z}}{\sqrt{2r{Rz}_{m}}L\left(2\alpha +\sin2\alpha \right)\sigma_{s}}-\frac{2F_{n}f_{z}}{\pi {\sqrt{2r{Rz}_{m}}L^{2}E}_{eq}}\right)\\ {Rz}_{b}={Rz}_{m}-\delta \end{array}\right.$$

where *Rzb* is the maximum roughness height after burnishing, *F_n_* is the force applied by each roller, *f_z_* is the wedge width that equals to the feed per each cutting insert, *Rz_m_* is the milling roughness height. For milling, *2α* can be obtained based on the geometry of the wedge. And *σ_s_* is the material flow stress.

However, when surface rolling is carried out on the surface with the milling condition #2, the roughness does not have the shape of a wedge and Equation (9) is no longer valid for the calculation of *Rzb*. In this condition, the asperities are deformed like a simple compression of a trapezoid with a height of Rz_m_ and width of *fz* (bigger) and *b* (smaller). Therefore, assuming it is an elastic-work hardening material, the stress–strain relationship and corresponding deformation can be obtained using the following equations.

(13) $$\sigma_{p}=K{\varepsilon_{p}}^{m},\;\;\;\sigma_{p}=\frac{F_{w}}{b},\;\;\;\varepsilon_{p}=ln\left(\frac{{Rz}_{m}}{{Rz}_{b}}\right),\;\;{Rz}_{b}={Rz}_{m}exp\left(-{\left[\frac{F_{w}}{bK}\right]}^{\frac{1}{m}}\right)$$

where *b* and *Fw* are the smaller width of the trapezoid and force applied to each roughness, respectively, that can be calculated using following equations. *K* denotes the Hollomon power law coefficient and *m* is the strain hardening exponent of the material.

(14) $$b=f_{z}-2\sqrt{2\left(a_{p}-{Rz}_{m}\right)r_{\varepsilon }-{\left(a_{p}-{Rz}_{m}\right)}^{2}}$$

(15) $$F_{w}=\frac{F_{n}f_{z}}{L\sqrt{2R{Rz}_{m}}}$$

When the surface rolling process is carried out at the multipass, it is assumed that a wedge shape is no longer appropriate for the SLM surface profile and milled surface profile under condition #1. Here, like explained above, the deformation of roughness is based on the compression of a trapezoid. Accordingly, as described in Equation (13), the smaller width of the trapezoid which is under compression and corresponding roughness height at further pass numbers can be obtained using the following formula:

(16) $$b_{n}=\frac{Rz-{Rzb}_{n}}{Rz}S_{m}\;\;\;\;\;{Rzb}_{n+1}={Rzb}_{n}\;exp\left(-{\left[\frac{F_{w}}{b_{n}K}\right]}^{\frac{1}{m}}\right),\;\;n=1,\;2,\;\ldots ,N$$

After calculation of the modified roughness height induced by burnishing, the surface profile follows the pattern of SLM or milling with modified Rz values; also, the Ra can be calculated using the following formula:

(17) $$Ra=\frac{1}{\lambda }\int_{0}^{\lambda }Z\left(X\right)dX$$

where λ is the preferred length based on the roughness cut-off distance.

### 2.2. Experimental Work

In order to confirm the results which were derived from an analytical model, a series of milling and surface rolling experiments were carried out on 3D-printed samples made of stainless steel 316L. the material was selected because of its superior printability and application in different industries [20]. The samples were selectively laser melted using an EOS 280 machine with a 1100 nm wavelength discontinuous Yb-fiber laser following the optimized standard conditions with a laser power of 195 W, scan speed of 1083 mm/s, 80 µm hatch spacing, and 20 µm laser thickness. The parameters were selected in such a way that the volumetric energy density reaches 100 J/mm^3^. The physical and mechanical properties of the 3D-printed material are provided in Table 1.

The 3D-printed samples were then subjected to the face milling process using a tool with three squared inserts with a nose radius of *r_ε_* = 0.8 mm and approach angle of *κ_r_* = 15°. The samples were face milled using a depth of cut of 0.2 mm with a spindle speed of 800 RPM and feed velocity of 200 mm/min. Then, the 3D-printed and machined samples were subjected to the surface rolling process with a tool comprising four rollers with a diameter of 4 mm and length of 10 mm. The multi-roller tool is installed to a universal milling machine head with a maximum power of 15 hp and spindle speed of 3000 rpm.

During the surface rolling, the static forces were measured using a 3-component force dynamometer KISTLER 9257B. Accordingly, the penetration of the tool into the material continues until achieving the exact value for the force, and then the rolling of the surface begins. Our preliminary experiments showed that the only surface rolling parameter that has significant effect on roughness change in multi-roller face surface rolling was static force. Therefore, the surface rolling experiments were carried out under different values of forces, i.e., 750 and 1500 N, and different pass numbers, i.e., 1 and 3. Also, other parameters like spindle speed and linear transverse velocity were kept constant at 800 RPM, 200 mm/min, respectively. Table 2 demonstrates the experimental plan. The ranges of process factors provided in Table 2 have been selected because of our simulation model to achieve a nanoscale surface finish and to identify the effectiveness of process factors more comprehensively. Moreover, our lab experience and previous research carried out on the same material gave us insight into how to make the experimental plan. Since the surface rolling spindle speed and linear velocity were known to be insignificant on roughness alternation, it was decided to set them as high as possible to obtain the minimum processing time. On the other hand, the pass number and force were first identified in our simulation model and then set on the machines and modified based on process limitations. Moreover, regarding the milling process factor, the spindle speed and feed rate was selected based on the limitation of the machine and cutting insert in terms of vibration and tool wear. Also, the depth of cut was set more than the roughness maximum height remaining from the SLM process to generate a new surface roughness.

The as-built specimens together with the milled and burnished samples were subjected to surface roughness measurements using a TylorHobson contact-based scanning machine and the 2D and 3D surface topographies; as well, their main roughness indices, i.e., Ra and Rz, were measured. During the measurement, the cut-off length for the as-built samples was set to 2.5 mm as the roughness values were between 2 and 10 µm; for milled and burnished samples, it was set at 0.8 mm because of the significant reduction in roughness.

For each set of experiments shown in Table 2, three runs were carried out and the average values of roughness have been reported in this paper. Also, the error bar has been provided based on the deviation from the average values. To compare the results driven by the simulation and the experimentally measured ones, the prediction error was defined as follows:

(18) $$Error\left(\%\right)=\frac{\left|Measured\;value-Simulated\;value\right|}{Measured\;value}\times 100$$

## 3. Results

**Figure 5:**
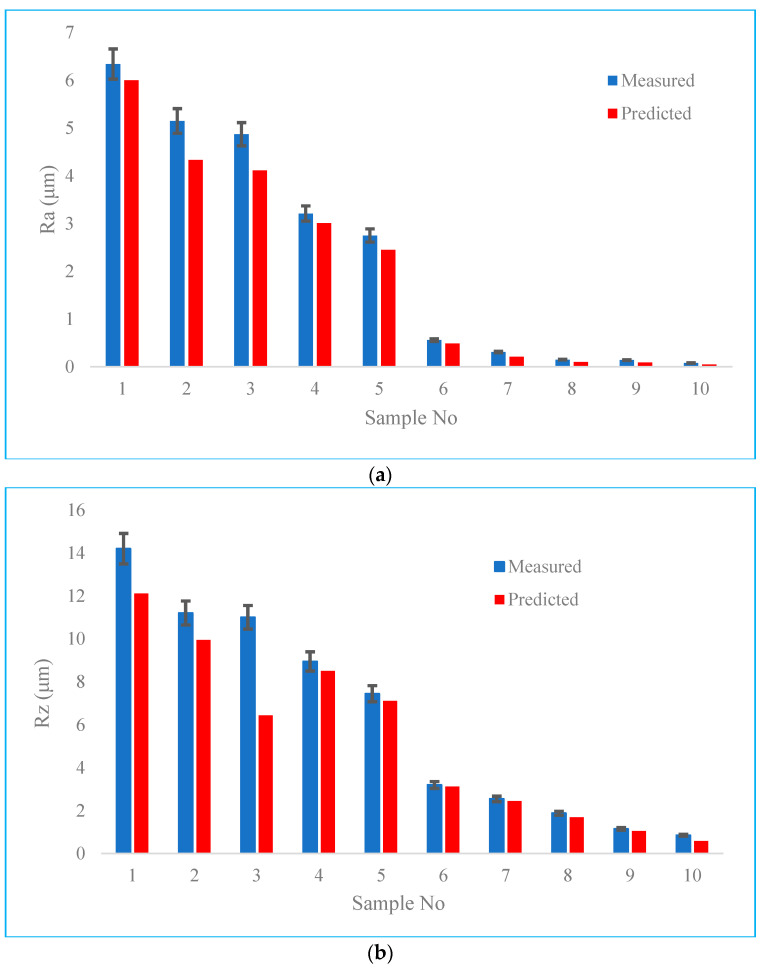
Comparison between the measured and predicted values of roughness for data sets of Table 2 (**a**) Ra (**b**) Rz.

To use the simulation framework as a practical tool, the derived values from the predictive model should be verified with confirmatory experiments. The obtained results regarding the values of arithmetic roughness *Ra* and maximum roughness height *Rz* have been presented in Figure 5. According to the figure, it is seen that there are compatible results between the measured values of roughness with those derived by the simulation framework.

The average prediction error for Ra is about 10%, while this value for Rz is around 7%. The results agree with previous work by this author, while a different approach for modeling of the surface roughness alternation was developed [21].

The variations in errors corresponding to the difference between the measured and predicted values are interesting, as shown in Figure 5. Accordingly, it is seen that the minimum prediction error is for data #6 that corresponds to the sample which is built by SLM and post-processed by milling. As the milling is a mechanical material removal process, the surface can be generated by having the geometry of contact and duplication of the engagement region following kinematics of motion. Moreover, as the milling is carried out at a finished machining regime, factors such as chatter vibration do not have a significant impact on the surface roughness; hence, neglecting this effect does not produce a significant error in our simulation model.

According to Figure 5, it can be inferred that following data number #6, the prediction error of samples 7 to 10 is lower than others. It can be attributed to the fact that the developed simulation model corresponding to these data sets is for the surface roughness values of two contact-based mechanical post-treatments, where their noise factors are minimized compared to melting-based processes. However, the main source of error in this batch of data is supposed to be due to neglecting the effect of surface elastic rebound that results in underestimating the prediction results.

The prediction error of data #1 corresponding to the as-built material is ranked #3 among the data batches. As the process follows melting and solidification, there are several noise factors which may cause errors while developing a predictive model. The source of error can be counted as neglecting the effect of inclusion as result of the formation of non-molten particles. However, this effect is not as significant as the main mechanism of roughness generation, i.e., cap formation over the melt pool surface.

The biggest values of prediction errors correspond to the samples which were post-processed by surface rolling immediately after SLM. In this condition, as the surface roughness of the as-built material is high enough, inducing a high amount of mechanical plastic deformation (that is not cutting), this results in the formation of scratches and flakes on the surface which were not considered in the simulation model. It is seen that nevertheless by increasing the pass number and static force, the surface roughness in both the simulation model and experimental values is being decreased, but the prediction error increases. It is attributed to the fact that increasing further the mechanical work (as a result of the bigger force and pass number) results in increasing the work hardening of the material. In such conditions, the material becomes more brittle and an excessive amount of load causes the formation of fractures in the material during plastic deformation. As this effect was neglected in our model, the error values in this batch of data are relatively higher than other data sets; however, they are still less than 10%, which is acceptable based on the values reported in other literatures.

**Figure 6:**
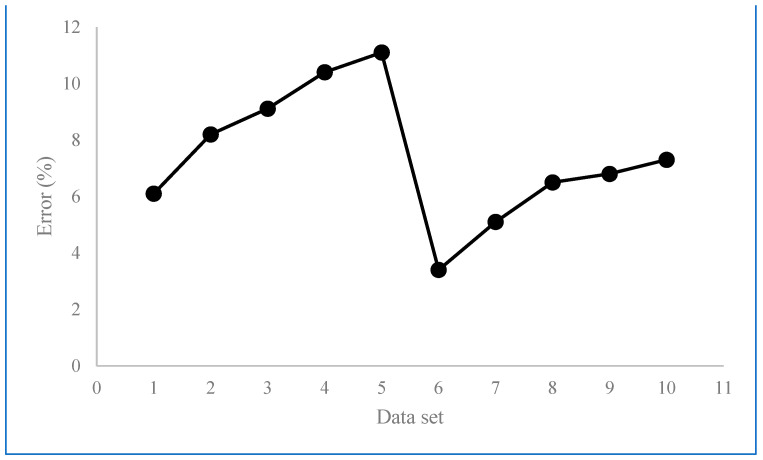
Variation of prediction errors for Ra of data sets.

Another point that is interpreted from Figure 6 is that regardless of the initial roughness which comes from either SLM or SLM + Milling, the prediction error increases when increasing the surface rolling force and pass number. As result of the increase in force and pass number, the work hardening and springback effects which were not considered in our model might be more emphasized. Accordingly, neglecting this fact may result in further prediction error.

## 4. Discussion

Once the model is verified by confirmatory experimental values of roughness, and the source of errors were identified, it can be used to analyze and influence process parameters for variations in surface roughness. Here, through this discussion we are going to identify how we can optimize the chain to achieve samples with the lowest surface roughness in shortest period of the time. Then, the evolution of the surface roughness through the best chain is identified and presented through 3D surface topographies.

**Figure 7:**
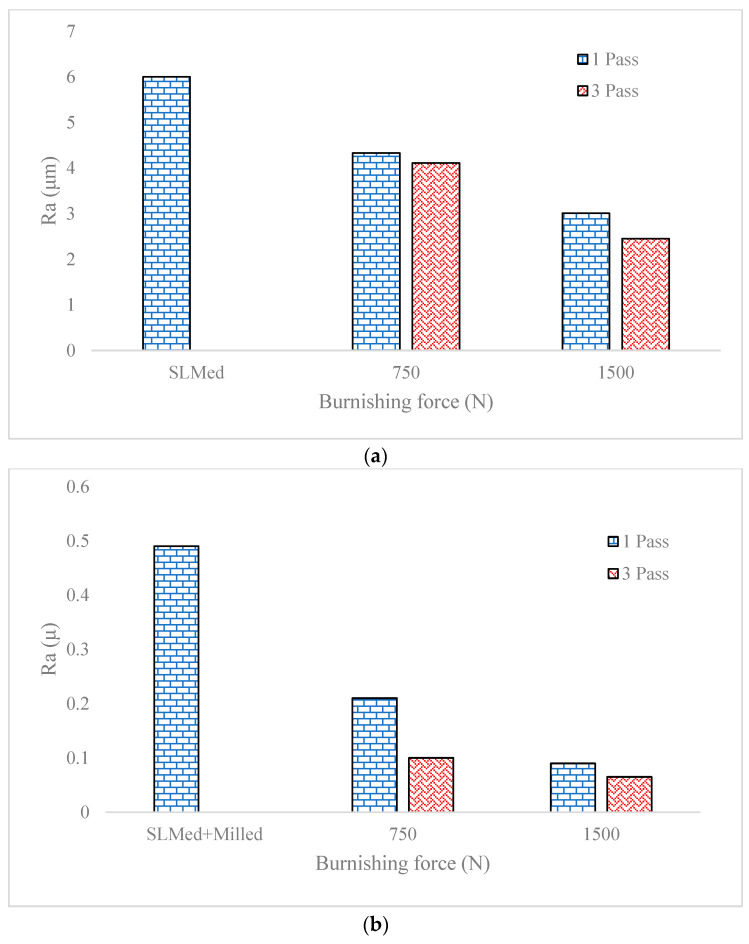
Variation of roughness under different values of rolling force and pass number for (**a**) SLMed samples (**b**) SLMed + Milled samples.

Figure 7 illustrates the interaction effect of the pass number and force when they are used for post-processing of an SLM sample (Figure 7a) and SLM + Milled sample (Figure 7b). According to Figure 7a, it is seen that when the process is carried out on an SLM sample, by increasing the pass number and static force the surface roughness of the as-built material decreases up to 60%. It means that the surface roughness of the as-built material from 6.38 µm reaches 2.45 µm when the force is 1500 N and the pass number is 3. It can be also seen that at a static force of 750 N, increasing the pass number does not have a significant influence on the roughness. However, when the static force reaches 1500 N, it is seen that the surface roughness decreases at further pass numbers. This effect can be attributed to the work hardening rate of the material at 750 N that results in no more reduction in roughness values at further passes, while the applied load is same. However, for bigger numbers of static loads, the surface roughness decreases by increasing the pass number and reaches 1.8 µm in the best conditions.

On the other hand, according to Figure 7b, it is seen that by applying the milling process after burnishing, the surface roughness reaches a value of 0.4 µm. It is seen that when a single-pass surface rolling with a static force of 750 N is carried out on the SLM + Milled sample, the roughness values reaches 0.15 µm. Moreover, it is seen that the increase in pass number and static force to 3 and 1500 N, respectively, results in a reduction in roughness but their effects are saturated and no more big changes in roughness values are achieved.

The production time is an important factor for decision making in a chain and can be calculated by sumation of the time of SLM, time of milling, and time of the surface rolling process. As the SLM time is fixed in the chain (all the materials are produced by same SLM setting), the production time is mainly determined by time of milling and surface rolling. On the basis of process kinematics, the milling and surface rolling times are calculated by dividing the length of the materials to be processed and linear velocity, i.e., *t* (min) = *L* (mm)/*V_f_* (mm/min). As the length of the workpiece is 70 mm and the linear velocity for both the milling and burnishing was set 200 mm/min, the time of processing for each individual operation is 21 s. For multi-pass surface rolling, this amount is multiplied for the number of surface rolling passes.

**Figure 8:**
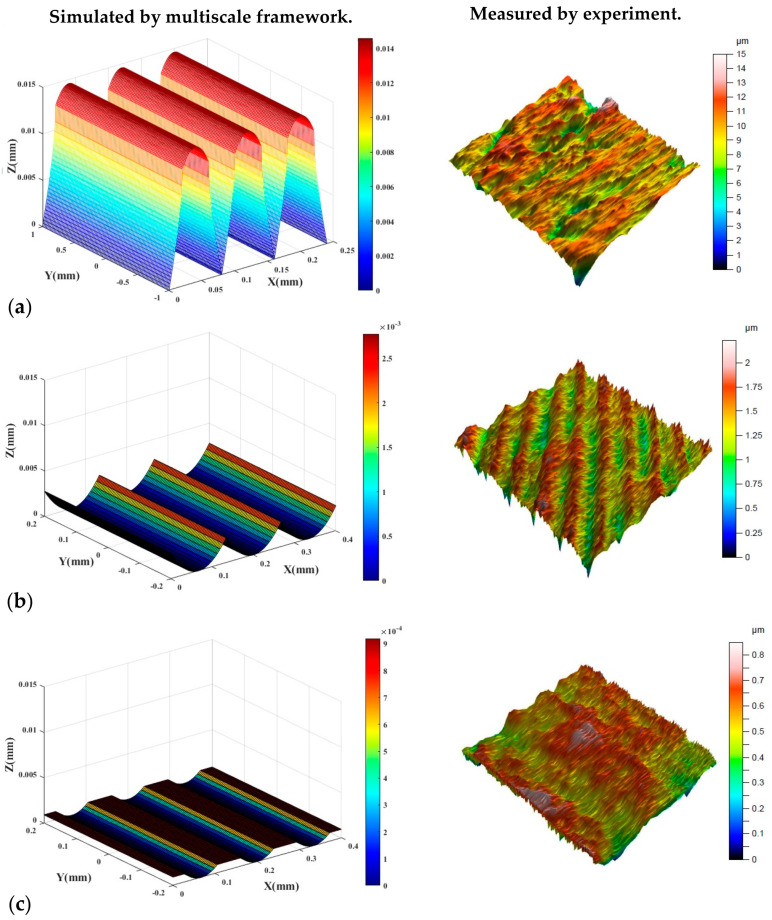
Alteration of simulated and measured optimum 3D surface topography in optimum chain design. (**a**) SLM sample, (**b**) milled sample, (**c**) burnished sample.

According to the abovementioned explanations, the optimized path which covers both criteria of the product’s quality and production rate (minimum production time) is when the SLM samples are post-processed by a single-pass milling and single-pass surface rolling with a static force of 750 N that results in a 42 s post-processing time. According to the simulation results, it is found that by eliminating the milling process, even when increasing the surface rolling time and force (that consumes lots of energy), the desirable surface roughness cannot be accessed. To better understand this finding, Figure 8 presents the variation in surface roughness under bigger values of force and roughness. It is seen that only by performing surface rolling with a force of 3000 N and 6 pass number (that equals 126 s post-processing time) are surface roughness values below 0.2 µm. However, setting this amount of force in practice is impossible and results in excessive tool wear and band breakage of rollers and the tooling system during the surface rolling process. Nevertheless, the burnishing pass can be performed as many times as possible, as has been reported in the literature [21,22]. However, inducing this amount of force for multiple burnishing passes may result in failure of the tool due to high friction, wear, and breakage. On the other hand, this work is going to show how a chain can be designed and optimized in the design stage as an advantage of developing simulation models.

Therefore, it can be inferred that applying a single milling process in a chain as middle operation between the SLM and surface rolling results in reducing the time of production by six times and the required force by three times to achieve same surface roughness. Accordingly, a process is added in the chain but it results in a significant reduction in the process time and the corresponding energy consumption that is function of time and force.

Figure 8 represents the evolution of the 3D surface topography for the optimum chain which is initiated by the SLM process and then post-processed by milling and burnishing. In the figure, both the simulated and measured surface topographies were included that confirm that the developed simulation framework can be used as a practical tool for the optimization of a chain including an AM process and following mechanical post-treatment.

## 5. Conclusions

An analytical simulation model has been presented to model the alternation in surface roughness that is generated by selective laser melting and post-processed by milling and surface rolling. Here, the surface is generated by the formation of caps over the melt pool in the SLM process and duplicated following laser head kinematics; then, the generated surface roughness is modified by milling and the generation of new roughness profile and surface rolling by flattening the roughness height. The model was verified from the surface roughness values of 10 samples built and post-processed under different experimental conditions. The goal of this research was to identify how planning of an optimal chain in the design stage can significantly optimize the product’s quality and minimize the production time. The obtained results show that:

- The obtained results which were derived from the simulation framework were compatible with the experimental values, while the average error for prediction of the arithmetic roughness was 10.1% and for prediction of the maximum distance between roughness peaks and valleys was 7.3%.
- It was found that the simulated surface roughness which is modeled in a chain of SLM + Milling + Surface rolling has a lower prediction error than the chain of SLM + Burnishing because of existing finished milling processes.
- It was found that by eliminating milling from the chain the production time will significantly increase by three times and further forces and energy are required to obtain same roughness. Therefore, elimination of a process from the chain does not guarantee minimizing the production time.

## Figures and Tables

**Table 1:** Physical and mechanical properties of SS316L [4].

Properties	Symbol	Unit	Values
Density	*ρ*	kg/m^3^	7800
Thermal conductivity	*k*	W/m°K	14
Specific heat	*C*	J/kg°K	460
Melting point	*Tm*	°K	1678
Absorptivity	*η*	-	0.35
Elasticity modulus	*E*	GPa	210
Yield strength	*σ_s_*	MPa	205
Strength coefficient	*K*	MPa	1356
Poisson ratio	*ν*	-	0.28
Strain hardening exponent	*m*	-	0.453

**Table 2:** Processed samples.

Sample No	Initial Condition	Rolling Static Force	Pass Number
1	SLM	Not applicable	Not applicable
2	SLM	750	1
3	SLM	750	3
4	SLM	1500	1
5	SLM	1500	3
6	SLM + Milling	Not applicable	Not applicable
7	SLM + Milling	750	1
8	SLM + Milling	1500	3
9	SLM + Milling	750	1
10	SLM + Milling	1500	3

## Data Availability

The data will be available upon request.
